# Economics of the clinical management of lung cancer in France: an analysis using a Markov model

**DOI:** 10.1038/sj.bjc.6601547

**Published:** 2004-01-20

**Authors:** C Chouaïd, L Molinier, C Combescure, J P Daurès, B Housset, A Vergnenègre

**Affiliations:** 1 Service de Pneumologie, Hôpital St Antoine, 184 rue du Fbg St Antoine, Paris Cedex 12 75571, France; 2 Laboratoire de Santé Publique et d'Epidémiologie, Inserm U558, Faculté de Médecine, Toulouse, France; 3 Institut Universitaire de recherche clinique, Faculté de Médecine de Montpellier, France; 4 Service de Pneumologie, Centre hospitalier Intercommunal, Créteil, France; 5 Service de l'information Médicale et de l'Evaluation, Service de Pneumologie, Hôpital Cluzeau Limoges, France

**Keywords:** Markov model, management, lung cancer, economic analysis

## Abstract

To evaluate, according to the histologic type and initial stage, the mean cost (MC) of managing patients with lung cancer and the costs of the different management phases. A Markov approach was used to model these costs, based on the management of a representative nation-wide sample of 428 patients with newly diagnosed lung cancer. The 18-month MC ranged from US$ 20 691 (95% CI: 5777–50 380 for diffuse non-small-cell lung cancer (NSCLC) to US$ 31 833 (95% CI: 15 866–64 455) for localised small-cell lung cancer (SCLC); first-line treatment costs ranged from 33.8% of MC for medically inoperable localised NSCLC to 74.6% for diffuse SCLC; second- or third-line treatment costs ranged from 7.8% of MC for surgically treated localised NSCLC to 32% for locally advanced NSCLC; and the cost of palliative care ranged from 9.1% of MC for locally advanced NSCLC to 39.9% for medically inoperable localised NSCLC. The cost of first-line chemotherapy and the percentage of actively treated patients impacted more on MC than did the cost of second- or third-line chemotherapy regimens or the cost of palliative care. In conclusion, this model provides a robust economic analysis of the cost of lung cancer management, and will be useful for assessing the economic consequences of future changes in patient management.

Lung cancer is one of the most serious public health problems in industrialised countries. Several studies have shown the high cost of this malignancy for health-care systems (Goodwin and Shepherd, 1998), leading to profound reflections in an era of health cost rationalisation (Berthelot *et al*, 2000; Bahl and Falk, 2001). Indeed, the incidence of lung cancer is increasing rapidly, and new costly antimitotic agents are yielding a moderate but significant survival increment. Previously, economic studies in this area are based on data extracted from randomised clinical trials and expert reports (Evans *et al*, 1995,^1997^; Evans, 1997; Earle and Evans, 1999) or are purely descriptive (Hillner *et al*, 1998; Wolstenholme and Whynes, 1999). Costly drugs used for the active treatment of lung cancer have also been the subject of cost-efficacy studies, generally comparing two alternative therapies and being limited to the initial treatment phase (Jaakkimainen *et al*, 1990; Goodwin *et al*, 1988).

These previous studies have several limitations. First, they were rarely based on routine management of a representative patient sample. Second, the models were based on expert opinion, which can differ somewhat from real practices. Third, data taken from clinical trials are unrepresentative, in terms of the patient population and management practices (Cottin *et al*, 1999). Finally, the management of lung cancer is becoming more complex, and the emergence of new antimitotic drugs calls for individual analysis of the respective costs of the different phases of patient management, especially those linked to first-line treatments, active second- or third-line treatments, and palliative care; costs must also be analysed according to the stage at diagnosis, which determines the chosen treatment strategy.

The aim of this study, based on the records of a sample of patients drawn from a French nationwide survey, was to assess the mean cost (MC) of the clinical management of lung cancer and the costs of the different management phases. We modelled these costs in order to assess the economic impact of the percentage of actively treated patients and the types of chemotherapy used.

## METHODS

Based on a sample of patients with lung cancer who were representative of the French national population of such patients, we examined management practices and their costs. A Markov model was used to calculate the MC of patient management according to the histologic type and extension stage at initial diagnosis, and the respective costs of the different treatment phases.

**Figure 1:**
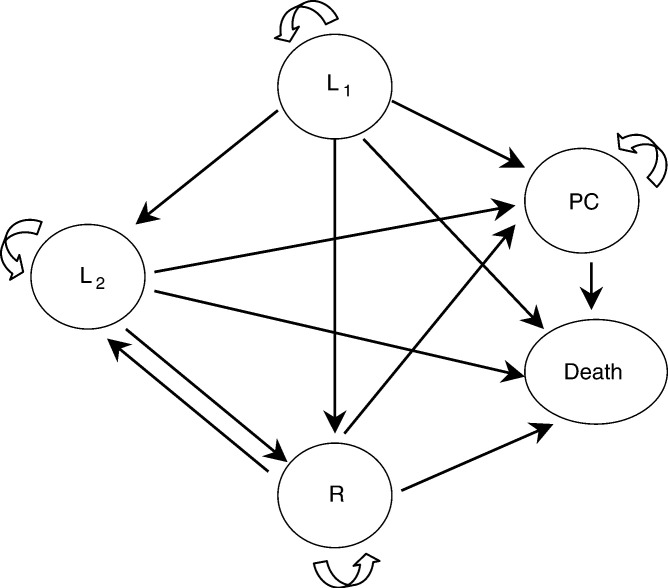
Modelling lung cancer management on the basis of five mutually exclusive and collectively exhaustive health states: L1=first-line treatment, L2=second- or third-line treatment, R=remission, PC=no active treatment and death. The arrows represent the possible transitions (arrows drawn to and from a given state denote the possibility of remaining in that state during a cycle).

The Markov model (Sonnenberg and Beck, 1993; Chouaid *et al*, 1998) is a multistate transitory model in which patients make transitions among various health states, at different rates, over extended periods. All clinically important events are modelled as transitions from one state to another. The passage of time is divided into intervals called cycles, which are chosen to represent a clinically meaningful time interval. During each cycle, each member of the cohort may remain in the same state of health or move to another state, except when the state is ‘absorbing’. In this way, lung cancer management can be modelled as follows: at diagnosis, the initial assessment (histologic type, stage, patient's age and general status) orients the management decision towards active treatment combining radiotherapy, chemotherapy and/or surgery, depending on the case (state L1: first-line treatment), or palliative care (state PC: no active treatment). When active treatment is undertaken, regular clinical assessment of efficacy and tolerability determines subsequent management, that is, pursuit of the same treatment (state L1); a second- or third-line treatment (state L2); simple monitoring for patients in partial or complete remission (state R); or palliative care for patients with disease progression (state PC). In other words, between each clinical assessment, each patient (Figure 1

) is in one of the following five health states: L1, L2, R, PC or death. These health states are mutually exclusive and collectively exhaustive.

### Primary data sources and identification of baseline and transition probabilities

Baseline probabilities and probabilities of transition from one state of health to another over time were established by analysing the management modalities of all consecutive new cases of lung cancer diagnosed between 1 July 1998 and 30 June 1999 in a representative French national sample of health-care centres managing lung cancer. In order to avoid including too many centres treating very few patients with potentially unrepresentative management approaches, only centres treating more than 50 cases of lung cancer annually were selected. One in 10 centres meeting this criterion were randomly selected after stratification, according to the type of establishment (in France, 30% of patients with lung cancer are treated in public university hospitals, 25% in nonuniversity public hospitals, 20% in cancer treatment centres and 25% in private establishments). On the basis of current international recommendations (Johnson, 1999; Hoffman *et al*, 2000), patients are categorised into seven homogeneous subgroups, two for small-cell lung cancer (SCLC) (localised and diffuse forms) and five for non-small-cell lung cancer (NSCLC), namely localised and initially operable, localised and medically inoperable, locally advanced receiving neoadjuvant treatment, inoperable locally advanced and diffuse forms. The sample size was calculated to obtain at least 20 patients in each subgroup. Patients managed in each institution were identified from individual medical data management systems (diagnosis-related groups, DRGs), by searching for all patients who consulted or were admitted to a medical or surgical ward with a principal diagnosis of lung cancer or suspected lung cancer, and by analysing all new radiotherapy-based treatments started during the study period. Patients treated for relapse and patients treated in clinical research trials were excluded. The analysis, which spanned the 18 months that followed diagnosis, or the period from diagnosis to death, focused on all events related to lung cancer that entailed the consumption of medical resources, including adverse effects of treatment necessitating hospitalisation, and patient transportation. Data were collected from patient charts by specially trained clinical research technicians. The physicians in charge of each patient were contacted to obtain data missing from the files. The exhaustive nature of patient enrollments and data collection in each centre were verified by two of the authors (LM and AV). We distinguished among the different management phases, for each patient and 3-month period, namely first-line treatments (surgery, chemotherapy or radiotherapy), second- or third-line treatment (all other active treatment periods), remission (periods in partial or complete remission) and palliative care, defined as a lack of conservative treatment (including palliative radiotherapy, anti-infectives, corticosteroids and pain relief). The patients were classified in each period by two of the authors (AV and CC). During each period, we also took into account costs linked to complications and patient transport. The clinical teams involved were informed of the study after it had been completed, to avoid influencing their practices.

### Economic valorisation

The economic analysis (Desky and Naglie, 1990) adopted the health-care payer's perspective and took into account only direct costs (i.e. consumption of health-care resources). Indirect costs (e.g. lost income) and intangible costs (e.g. pain and suffering) were not assessed. Direct costs of managing lung cancer included hospitalisation, medical costs and transportation. Hospitalisation costs (administration, security, maintenance, general equipment, central supply, dietetics and social services) were assessed on a per diem basis (national unit cost scale for each event) for fixed costs and on drug purchase prices in the establishments concerned. Depending on the reason for hospital admission and the length of stay, the mean unit cost per hospital day ranged from 202US$ (day-care radiotherapy) to 656US$ (surgery). Medical procedures performed outside the hospital and transport costs were assessed using the national unit cost scale. Medical costs (nursing, care, ward supplies, pharmacy, diagnostic tests, laboratory tests and professional services) were determined retrospectively by chart review. The volumes of resources used were identified in each 3-month period by distinguishing the consumption linked to surgery, chemotherapy, radiotherapy, treatment complications, monitoring, palliative care and transportation. The initial diagnostic costs were not taken into account.

### Markov model

Using Decision Analysis software from TreeAge® Data 3.5. (Williamstown, MA, USA), we analysed the expected monetary cost of going through the Markov model. The simulation was run as follows. In each subgroup, patients were distributed between the different health states according to the initial probability (P0) of being treated (state L1) and, therefore, the probability (1-P0) of receiving palliative care (state PC). In the subsequent cycle (3-month period), the cohort was partitioned among all the states according to the transition probabilities; this resulted in a new distribution of the cohort among the different states. The utility accrued for the cycle is referred to as the cycle sum. In total, 95% confidence intervals (CI) were obtained by Monte-Carlo simulation. Groups were compared using Student's *t*-test (Thompson and Barber, 2000), run on Statview® 4.02 (Abacus Concept Inc., USA).

### Sensitivity analysis

Several sensitivity analyses were conducted to test the relevance of the model, first, by varying, in the relevant subgroups (diffuse SCLC, inoperable locally advanced and diffuse NSCLC), the percentage of actively treated patients (initial probability of treatment, P0) from 75 to 100%; then by studying the impact of the costs of chemotherapy regimens used in first- and second- or third-line treatments, by varying these costs by ±30%; and finally by taking into account new management practices in palliative care (home-based care), by varying these costs by −30%. These three analyses were applied to each subgroup.

## RESULTS

### Baseline data

The study included 428 patients (Table 1

**Table 1:** Characteristics, initial stage and management modalities

**Histology**		**NSCLC**				**SCLC**	
**Forms, number of patients**	**Local Op, 58**	**Local Nop, 22**	**LA-Ad, 21**	**LA-Nad, 99**	**Diffuse, 140**	**Local, 36**	**Diffuse, 52**
Age	70±11.2	59±11.4	55±9.2	63±11.3	55±9.2	63 (10.9)	63±9.5
Death 18 months	14 (24.1%)	14 (64%)	8 (38%)	69 (70%)	125 (89%)	23 (64%)	48 (92.3%)
Management							
PC	0	0	0	1 (1%)	4 (2.9%)	0	2 (3.8%)
*L1*	58 (100%)	22 (100%)	22 (100%)	98 (99%)	136 (97.1%)	22 (100%)	50 (96.2%)
Surgery only	19	—	—	—	—	—	—
Surgery plus	39	—	11	—	—	—	—
Radiation	—	12	—	10	—	—	—
Chemotherapy	—	5	9	65	124	9	40
Radiochemotherapy	—	5	1	23	12	27	10
							
*L2*	17 (29.3%)	5 (22.7%)	18 (85.7%)	61 (61.6%)	58 (41.4%)	13 (36.1%)	18 (34.6%)
Radiation	—	—	6	16	3	—	—
Chemotherapy	15	5	4	17	50	9	13
Radiochemotherapy	2	—	6	30	5	4	5

NSCLC=non-small-cell lung cancer; SCLC=small-cell lung cancer; PC=no active treatment; L1=first-line treatment; L2=second- or third-line treatment; Local Op=localised, initially operated; Local Nop=localised, medically inoperable; LA-Ad=locally advanced, neoadjuvant treatment; LA-Nad=locally advanced, no surgery; diffuse=disseminated; surgery plus=surgery combined with chemotherapy or radiotherapy.

). The mean age was 61±3 years and the male–female sex ratio was 4.66. All the patients had a histologic or cytologic diagnosis, and the study population was representative of the epidemiology of lung cancer in France, with SCLC in 20.6% of cases (diffuse in 59%) and NSCLC in 79.4%. Two patients with disseminated SCLC (3.8%), four patients with disseminated NSCLC (2.9%) and one patient with locally advanced NSCLC (1%) received palliative care from the outset. In all other cases, management combined surgery (initially for localised forms, *n*=58, or after neoadjuvant treatment, *n*=11) and/or radiotherapy and/or chemotherapy (Table 1). With the exception of patients operated on initially, all the patients can be classified, at a given moment, in one of the following five health states: L1, L2, R, PC or death. Initially operated patients were distributed among six different health states, namely postoperative monitoring (POM), L1, L2, R, PC and death. As management was standardised in most cases, this classification was relatively straightforward. The MC of each management modality per 3-month period is summarised in Table 2

**Table 2:** Mean cost (US $) of each management modality, per 3-month period, according to the histologic type and stage

	**Cost (US $)**	**Chemotherapy (%)**	**Radiotherapy (%)**	**Surgery (%)**	**Complications (%)**	**Monitoring (%)**	**TC (%)**	**Transport (%)**
*Loc Op NSCLC (n=58)*								
SOP	5352	0	0	93.5	0.1	4.8	0	1.6
L1	14 812	22.4	19.6	44	7.5	0.5	0	6
L2	8976	33.6	23.6	17.4	13.7	0.8	0	10.9
R	542	0	0	0	0	86.9	0	13.1
PC	5801	0	0	0	0	0	88.9	11.1
*Loc-Nop NSCLC (n=22)*								
L1	8725	28.8	41.6	0	14.4	0.6	0	14.6
L2	6810	26.9	44	0	18.8	1	0	9.3
R	381	0	0	0	0	80.8	0	19.2
PC	8315	0	0	0	0	0	89.2	10.8
*LA-Ad NSCLC (n=21)*								
L1	15 918	27.4	10.9	51	3.1	0.1	0	7.5
L2	6162	55.8	21.6	5.5	2.9	0.8	0	13.4
R	484	0	0	0	0	88.8	0	11.2
PC	2982	0	0	0	0	0	96.3	3.7
*LA-Nad NSCLC (n=99)*								
L1	10 424	46.9	29.3	0.6	10.5	0.2	1.5	11
L2	6790	40.6	27.6	5	5.8	0.6	9.3	11.1
R	554	0	0	0	0	90.9	0	9.1
PC	7140	0	0	0	0	0.5	95.2	4.3
*Distant NSCLC (n=140)*								
L1	10 476	57	10	6.6	13.7	0.9	5.8	6
L2	6555	65.9	10.1	1.8	14.7	1.9	0	5.6
R	539	0	0	0	0	84.5	0	15.5
PC	5764	0	0	0	0	0.3	92.8	6.9
								
*Local SCLC (n=36)*								
L1	12 436	42	27.9	1.6	14.9	2	0	11.6
L2	7311	62.5	13.6	0	11.1	5	0	7.7
R	684	0	0	0	25.4	59.5	0	15.1
PC	5279	0	0	0	0	0.6	85.7	13.7
*Distant SCLC (n=52)*								
L1	10 718	52.1	8.8	3	26	4.1	0	6
L2	6100	72.2	7.6	0	5	7.4	1.3	6.5
R	596	0	0	0	0	89.4	0	10.6
PC	3521	0	0	0	0	0	90.4	9.6

NSCLC=non-small-cell lung cancer; SCLC=small-cell lung cancer; PC=no active treatment; L1=first-line treatment; L2=second- or third-line treatment; POM=postoperative monitoring; R=remission; PC=no active treatment; Local Op=Localised, initially operated; Local Nop=localised, medically inoperable; LA-Ad=locally advanced, neoadjuvant treatment; A-Nad locally advanced nonsurgical; Distant=disseminated; TC=terminal care.

. In the case of medically inoperable localised NSCLC, the costs of periods L1, L2 and PC were equivalent. In the other cases, period L1 always generated the highest costs. The cost of period L2 ranged from 38.7 to 65.1% of the cost of period L1, and the cost of period PC ranged from 18.7 to 68.4% of the cost of period L1. The cost breakdown for the periods of active treatment (L1 and L2) varied considerably according to the subgroup: the cost of chemotherapy predominated in SCLC and in locally advanced and disseminated NSCLC; the cost of radiotherapy predominated in medically inoperable localised NSCLC; and surgery was the principal cost in operable NSCLC. Treatment complications represented up to 26% of the costs, and transportation accounted for 6–15.1%.

### Application of the Markov model

The patient distributions, in each subgroup per 3-month period, in the different health states determined the values of baseline and transition probabilities and their changes with time (data not shown). Running the Markov model for six cycles, and using Monte-Carlo simulation, the MC at 18 months was, respectively, US$ 20 184 (95% CI: 3521–46393) and 31833 (95% CI: 15 866–64455) for diffuse and localised forms of SCLC. In NSCLC, it ranged from US$ 20 691 (95%: CI 5777–50380) for diffuse forms to US$ 27 794 (95% CI: 15918–25062) for locally advanced forms treated with neoadjuvant therapy (Table 3

**Table 3:** Mean cost (US$), standard deviation and 95% confidence intervals (CI) of 18 months of management, and the cost of each management phase

	**Mean cost (SD; CI)**	**L1**	**L2**	**R**	**PC**	
Local Nop NSCLC	24 443 (8650; 8327–48 846)	8277 (33.8%)	5924 (24.2%)	503 (2.1%)	9739 (39.9%)	
LA-Ad NSCLC	27 794 (3146; 15 918–25 062)	15 919 (57.3%)	8895 (32%)	442 (1.6%)	2538 (9.1%)	
LA-Nad NSCLC	24 579 (9327; 7140–52 341)	14 817 (60.3%)	4895 (19.9%)	607 (2.5%)	4260 (17.3%)	
Diffuse NSCLC	20 691 (9063; 5777–50 380)	12 913 (62.4%)	2714 (13.1%)	305 (1.5%)	4759 (23%)	
Local SCLC	31 833 (9984; 15 866–64 455)	21 549 (67.7%)	4831 (15.1%)	1331 (4.2%)	4122 (12.9%)	
Diffuse SCLC	20 184 (7309; 3521–46 393)	15 058 (74.6%)	2091 (10.4%)	318 (1.6%)	2718 (13.4%)	
						
	**Mean cost (SD; CI)**	**POM**	**L1**	**L2**	**R**	**PC**
Local Op NSCLC	25 050 (9718; 10 789–61 930)	6840 (27.2%)	12 625 (50.2%)	1803 (7.8%)	896 (3.6%)	2886 (11.2%)

NSCLC=no small-cell lung cancer; SCLC=small-cell lung cancer; PC=no active treatment; L1=first-line treatment; L2=second- or third-line treatment; POM=postoperative monitoring; R=remission; PC=no active treatment, Local Op=Localised, initially operated; Local Nop=localised, medically inoperable; LA-Ad=locally advanced, neoadjuvant treatment; LA-Nad locally advanced nonsurgical; distant=disseminated.

). Differences between the groups in both the mean and median costs (Table 3) were significant (*P*<0.001). Regardless of the subgroup considered, the standard deviations were very large, reflecting the dispersion of costs (linked to early death, complications requiring lengthy hospitalisation and lengthy management in palliative care units). The costs of the different phases varied significantly (*P*<0.001) according to the histologic type and stage at diagnosis. First-line treatment costs ranged from 33.8% of MC for medically inoperable localised NSCLC to 74.6% for diffuse SCLC; second- or third-line treatment costs ranged from 7.8% of MC for surgically treated localised NSCLC to 32% for locally advanced NSCLC treated with neoadjuvant therapy; and the cost of palliative care ranged from 9.1% of MC for locally advanced NSCLC to 39.9% for medically inoperable localised NSCLC.

### Sensitivity analysis

When the percentage of actively treated patients was increased from 75 to 100%, the MC of diffuse SCLC, and of inoperable locally advanced and diffuse NSCLC increased by 1.5, 0.8 and 0.76%, respectively, per 1% increment in the proportion of actively treated patients. The cost of chemotherapy used in first-line treatments had the largest impact on MC of SCLC and nonlocalised NSCLC; the cost of chemotherapy used in second- or third-line treatments mainly impacted on the mean cost of locally advanced NSCLC, and the cost of palliative care mainly impacted on the mean cost of medically inoperable localised and diffuse forms of NSCLC (Table 4

**Table 4:** Impact on the mean management cost of a lung cancer patient of a ±30% variation in first-line (L1) chemotherapy costs and second- or third-line (L2) chemotherapy costs and a 30% fall in palliative care (PC) costs

	**Mean cost (US$)**	**L1 chemotherapy costs (%)**	**L2 chemotherapy costs (%)**	**PC costs (%)**
Variation		±30	±30	−30
Local Op NSCLC	25 050	±3.5	±1.1	−4
Local Nop NSCLC	24 443	±2.9	±2.7	−13.6
LA-Ad NSCLC	27 794	±6.5	±3.1	−2.9
LA-Nad NSCLC	24 579	±8.4	±4.9	−6.1
Diffuse NSCLC	20 691	±10.6	±2.7	−6.9
Local SCLC	31 833	±8.5	±2.7	−4.2
Diffuse SCLC	20 184	±11.7	±2.3	−4.2

). The percentage of actively treated patients and the cost of chemotherapy regimens used in first-line treatments impacted more on MC than did the cost of chemotherapy used in second- or third-line treatments, or the cost of palliative care.

## DISCUSSION

This study shows that, during the first 18 months after diagnosis of NSCLC and SCLC, patient management costs an average of US$ 23 262 and 27 067, respectively. As previously reported (Wolstenholme and Whynes, 1999), the standard deviations and large CI reflected the existence of two subgroups of patients, one of which generated major costs (usually due to lengthy hospitalisation) and the other only minor costs (owing to early death). Extrapolating these sums to the entire French population, the cost of managing lung cancer is thus US$0.53 billion yearly (0.4% of all health-care spending). This model, based on a representative national sample of patients with lung cancer, takes into account the main factors influencing management costs, namely treatment modalities (hospital-based or ambulatory care), refunding (private or public sector) and health-care practices. This approach permits a robust economic analysis reflecting the reality of patient management and is a useful adjunct to previously published studies in this area, be they purely descriptive (Goodwin and Shepherd, 1998) or model-based (Evans *et al*, 1995).

Regarding NSCLC, some authors have attempted to estimate the cost of patient management without the use of models (Hillner *et al*, 1998; Wolstenholme and Whynes, 1999). Unfortunately, the aims, methodology, data collection modalities and types of costs examined differ markedly among these studies, making it difficult to compare the results. The most ambitious and most comprehensive model-based study is that published by Evans *et al* (1995), based on 1988 cost calculations. This work was regularly updated to take into account practice changes (Evans *et al*, 1997; Evans, 1997, Earle and Evans, 1999), particularly by examining the impact of neoadjuvant treatment for stage IIIA and chemoradiotherapy for stage IIIB. Using the same model, and considering that 85% of these patients received active treatment, the authors concluded that these first-line treatments increased the mean 1-year cost of patient management by 45.9–66.3% for stage IIIA disease and by 27.9% for stage IIIB disease. It should be stressed that this model does not take into account the cost of treatment-related complications or transport, which represented up to 26 and 15%, respectively, of total costs per patient in our study. In our study, first-line treatments represented, respectively, 57.3 and 60.3% of the MC of managing these forms of NSCLC. For stage IV NSCL (Earle and Evans, 1999), these authors estimated the MC of patient management with the paclitaxel–cisplatin combination at 44 756 Canadian dollars, that is, almost twice the cost that we calculated in France (US$ 20 184); but the Canadians postulated that all patients received first-line treatment with this costly combination.

Most importantly, our results are based on routine management of a representative sample of newly diagnosed lung cancer patients rather than on expert opinion, which does not necessarily reflect actual practices. Thus, in our study, 48 and 25.9% of patients with stage I and II NSCLC had a medical contraindication to surgery; 67% of those initially treated surgically and 99.2% of those with locally advanced disease were actively treated with chemotherapy and/or radiotherapy. In contrast, the corresponding proportions in the Canadian model were 10, 15, 20 and 85%.

Regarding SCLC, interpretation of the available costing literature is hindered by problems of variable cost inclusions, health systems and data expression (Graham and Boyages, 1993; Doyle *et al*, 1996). Even if the management of SCLC is more consensual than that of NSCLC, several studies confirm the broad range of chemotherapy protocols prescribed (Sambrook and Girling, 2001). Despite the lack of recommendations in this area, 35.2% of patients with SCLC in our study received second-line treatment, while the Canadian assumption (Evans *et al*, 1995) was that no second-line treatment was given to such patients. In contrast, prophylactic cerebral irradiation was used far less often in our population (33% of localised forms and 0% of diffuse forms, compared to, respectively, 70 and 30% in the Canadian model). An English team recently published the most thorough work to date (Oliver *et al*, 2001), consisting of a purely descriptive study limited to two hospitals. It did not distinguish diffuse and localised forms, and analysed 109 consecutive patients diagnosed with SCLC between 1994 and 1997 (91.7 and 17.4% of patients, respectively, received first- and second-line treatment). The MC per patient was £11 556 and, if one excludes the cost of diagnosis (£2022), phases L1, L2 and PC represented, respectively, 59.2, 9.2 and 23.9% of the total cost values similar to those we obtained.

The advantage of the Markov approach is that it can model all the items composing the cost of managing patients with lung cancer, especially the percentage of actively treated patients, drug costs and the number of chemotherapy cycles (Smith *et al*, 2001). This is a major advantage, given the increasingly complex management of lung cancer. The Markov model can also be used to evaluate the economic impact of new antimitiotic drugs (which are generally far more costly than reference treatments) and also takes into account new modes of drug administration (in particular, the use of oral treatments, which do not necessitate hospitalisation and therefore reduce direct costs). Finally, new palliative care structures, and especially home-based care (which is far less costly than hospitalisation), would engender a moderate cost reduction.

Our study has certain limitations. Being centred on the economic consequences of patient management choices, the cost of diagnosis (which is not influenced by treatment choices) was not taken into account. In previous studies (Evans *et al*, 1995; Oliver *et al*, 2001), these costs represented between 5 and 10% of the total management costs. Moreover, our economic assessment only covered the 18-month period following diagnosis. This is longer than in most studies, which generally focused on initial management costs or the first year after diagnosis, a period that generates between 85 and 97% of total costs (Evans *et al*, 1995; Oliver *et al*, 2001). The risk is that major costs are associated with a few surviving patients who receive lengthy palliative care. The Markov approach limits this weakness by allowing longer periods to be modelled, using the MC of periods spent with palliative care. Likewise, the Markov model can take into account late relapses of surgically treated NSCLC by studying 20 cycles (corresponding to 5 years).

Our sample took into account the existence of both public and private health insurance systems in France, and the type of health-care centre. However, by limiting our sample to centres managing more than 50 new cases annually, we induced a certain selection of the population and practices, which probably led to an overestimation of the proportion of actively treated patients. Nonetheless, the universal medical insurance system in France and the management of most cancer patients by specialists tend to favour more aggressive management. We excluded patients treated in clinical trials, in which costs are unknown, although a recent study showed little difference from routine clinical practice (Bennett *et al*, 2000).

Another limitation of our study was the outcome measure. Indeed, we limited our analysis to financial costs, without seeking to determine the utility of the different health states in which patients find themselves during the course of their illness. Such analyses are most useful for comparing therapeutic strategies (Arikian *et al*, 1995), whereas our objective was to determine the relative costs of the different phases of patient management.

## CONCLUSION

The Markov model used in this study, based on actual lung cancer management practices in France, yielded a precise cost analysis of the different management phases of lung cancer patients, and an assessment of the likely impact of future advances that are essential for the effective management of chronic diseases such as lung cancer.
